# Printability, Thermal and Compressive Strength Properties of Cementitious Materials: A Comparative Study with Silica Fume and Limestone

**DOI:** 10.3390/ma15238607

**Published:** 2022-12-02

**Authors:** Dodda Srinivas, Dhrutiman Dey, Biranchi Panda, Thallak G. Sitharam

**Affiliations:** 1 Centre for Intelligent Cyber Physical Systems, Indian Institute of Technology Guwahati, Guwahati 781039, India; 2 Sustainable Resources for Additive Manufacturing (SreAM) Laboratory, Department of Mechanical Engineering, Indian Institute of Technology Guwahati, Guwahati 781039, India; 3 Department of Civil Engineering, Indian Institute of Technology Guwahati, Guwahati 781039, India

**Keywords:** 3D concrete printing, extrusion, yield stress, cement and phase change materials

## Abstract

Over the past decade, 3D printing in the construction industry has received worldwide attention and developed rapidly. The research and development of cement and concrete products has also become quite well-established over the years, while other sustainable materials receive considerably lower attention in comparison. This study aims to investigate the influence of the two most commonly used sustainable cementitious materials i.e., silica fume and limestone powder, on printability, thermal and mechanical properties of fly ash–Portland cement blends. Ternary blends containing Portland cement, fly ash and silica fume or limestone powder are prepared, whereas phase change material (PCM) is introduced to improve the thermal behavior. Based on the rheological properties and concurrent 3D concrete printing, improved buildability of the modified mixtures is linked to their static yield stress. Anisotropic mechanical properties are observed for 3D printed specimens, while cast specimens exhibit a maximum 41% higher compressive strength due to better material compaction. It is clear from the results that addition of silica fume and limestone powder ranged from 5% to 10%, reducing the anisotropic mechanical properties (maximum 71% and 68% reduction in anisotropic factor, respectively) in the printed specimens. The PCM addition ranged from 5% to 10% and improved thermal performance of the mixtures, as measured by a decrease in thermal conductivity (9% and 13%) and an increase in volumetric heat capacity (9% and 10%), respectively. However, the PCM-containing mixtures show around 29% reduction in compressive strength, compared to the control specimen, which necessitates new material design considering matrix strengthening methods.

## 1. Introduction

3D Concrete Printing (3DCP) is one of the disruptive technologies for faster and form work-free construction. Recent developments in the field, such as 3D printed modular houses, pedestrian foot bridges, office buildings, public schools, low-cost toilet units etc., demonstrate the potential of initiating a paradigm change in the practice of construction [1,2]. These approaches vary in scale and comprise applications of both on-site manufacturing and prefabrication in plants that operate under a controlled environment. In contrast to the conventional casting, 3DCP requires time-dependent rheological properties; therefore, the rheological properties are important to understand the flow behavior of the concrete in its fresh state [3]. A printable concrete must have low yield stress for extrusion and high yield stress after the extrusion or deposition to retain its shape and prevent it from slumping [4]. In general, 3DCP mixtures require two to three times higher binder content than conventional concrete mixes and lack adequate thermal performance. Several efforts have been made to reduce the Portland cement content in the mix design via the use of supplementary cementitious materials (SCMs), such as fly ash (FA), silica fume (SF), and limestone powder (LP) as a partial substitution to cement [5]. Other industrial wastes, such as volcanic pumice dust, cement kiln dust and agro-wastes have also been shown to make environmentally-friendly concretes with improved properties [6,7].

In India, utilization of limestone has added the advantages of being abundant and inexpensive, which can further reduce the negative environmental impact caused by Portland cement. Limestone additives have demonstrated improvement in both the fresh and hardened properties due to synergetic effects of improved particle packing, increased hydration via filler effect, and the production of carboaluminate phases [8,9,10,11]. When employed in mixtures with PC as the key binder, limestone fillers are generally perceived as inert. However, when combined with supplementary cementitious materials, LP reacts with the calcium aluminate hydrates generated by the Pozzolanic reaction to produce hemi and mono carboaluminate hydrates [12,13,14]. This causes the ettringite to become more stable and will increase the volume of all hydration products, which may eventually lead to a reduction in porosity and an increase in strength [12,13,15]. Tao et al. [16] examined the effect of LP on the fresh and hardened properties of 3D printable mixes and concluded that high volume substitution (25% and 50%) of binder with LP had a detrimental effect. Skibicki et al. [17] showed in a study that while LP had no effect on the compressive strength of printable mixtures with higher binder contents, it was effective in improving the buildability and compressive strength of mixtures with lower binder contents. The primary benefit of using LP is that it not only enhances rheological performance, but also lowers the need for cement, making printable mixes more sustainable [18]. Similarly, the incorporation of SF has been reported to increase the yield stress and plastic viscosity of cementitious materials, while reducing the flowability. However, with different superplasticizer kinds and water-to-binder ratios, the SF might have diverse outcomes [19]. Kazemian et al. [20] found that 10% (by mass) addition of SF improved the fresh properties of the mixture. Authors were able to print a five-layered specimen using SF and found perfect shape stability and no visible deformation during the printing. Panda et al. [21] reported that SF had a significant impact on the rheology of geopolymer mixes. Using SF to replace 5–10% of the binder content nearly doubles the yield stress. Furthermore, the viscosity recovery was found to be 5–7% greater than the control mixture. It has also been reported that when used with magnesium potassium phosphate cement as a coating, the addition of SF improves the corrosion resistance of steel exposed to harsh environments [22].

In terms of thermal conductivity, porosity in the concrete is one of the major factors that impacts the overall behavior. The apparent density of concrete is, therefore, proportional to its thermal conductivity [23,24,25,26]. The rate of heat transfer and its capacity to store heat are determined by thermal diffusivity and volumetric heat capacity, in addition to thermal conductivity which is equally important for understanding the thermal behavior of a mixture design [27]. In a recent study, Brookes et al. [28] investigated the effect of microencapsulated phase change material (mPCM) on printability, mechanical, and thermal characteristics of 3D printable cementitious materials. Based on its physical attributes and volume loading, mPCM was discovered to alter the printability. Cui et al. [29] also employed mPCM into the 3D printable mixture in a certain percentage, such that it did not affect the material buildability and evaluated its adverse effect on the mechanical properties. In another study, Hao et al. [30] found a new shape-stabilized PCM for 3D concrete printing by impregnating paraffin into the recycled fine aggregates, and found that the thermal conductivity of printed concrete has a good correlation with dry density and porosity.

Despite significant research advances in material design, only a few studies have investigated the thermal behavior of 3D printed concrete [31,32]. In this regard, the use of phase change materials (PCMs) has been steadily increasing because of their capacity to store and dissipate energy in the form of heat. The ability to store and release heat also depends on the pore volume, sizes, and their distribution in the cementitious system which could be impacted by the incorporation of PCMs. The applications described above require concrete to demonstrate adequate printability (extrudability and buildability) as well as thermo-mechanical properties; therefore, this paper first compares rheological properties of ternary mixtures containing Portland cement, FA and SF or LP, with special emphasis on examining the influence of microencapsulated PCMs on thermal and mechanical properties. After 28 days of ambient curing, printed specimens are tested for determination of compressive strength, followed by anisotropic coefficient and compared to control specimens. Further, mold casted specimens are prepared, and their compressive and thermal properties are compared to those of printed specimens. The results from this study are expected to shed light on the printability, thermo-mechanical properties of cementitious materials and facilitate the choice of materials used in 3DCP.

## 2. Materials and Methods

### 2.1. Materials and Mix Preparation

**Figure 1:**
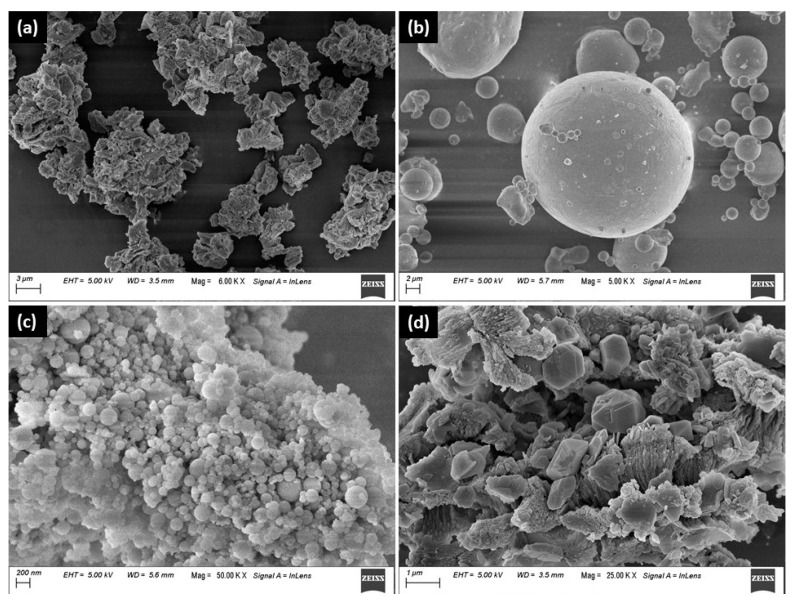
Morphology of (**a**) OPC, (**b**) FA, (**c**) SF, and (**d**) LP.

**Figure 2:**
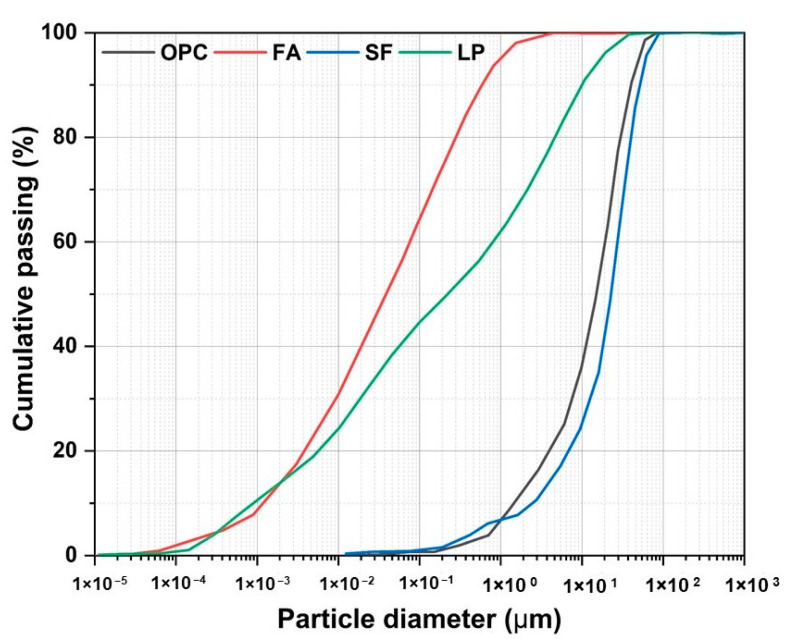
Particle size distributions of OPC, FA, SF and LP.

A 43-grade ordinary Portland cement (PC) (Dalmia Bharat Ltd., Thangskai, India) in accordance with the requirements of IS 8112: 2013; class F fly ash (FA) confirming to IS 3812 Part 1: 2013; densified silica fume (SF) supplied by Elkem Ltd. (Hyderabad, India); and limestone powder (LP) (1% residue at 200 mesh) were used as binders (b) in this study. Figure 1 and Table 1 show the morphology and chemical composition of these binders, respectively. A laser diffraction particle size analyzer (Malvern Mastersizer 2000, Malvern, UK) was used to measure their particle size distributions, and the results are shown in Figure 2. Methyl cellulose-based viscosity modifying admixture (VMA), in powder form, was added (0.1 wt.% of binder) to tailor the rheology of the mixes. Zone II river sand (s), with a maximum particle size of 2.36 mm confirming to IS 383: 2016, was considered as fine aggregate in this paper having specific gravity and fineness modulus of 2.65 and 2.75, respectively. CrodaTherm^TM^ ME29P—a commercially available, acrylic core shell (formaldehyde-free) powdered micro encapsulated PCM, with its core derived from plant-based feedstocks—was used, with a median particle size of <150 μm, density around 337 kg/m^3^ and melting temperature of 28.8 °C.

Ternary blends were prepared wherein: PC was replaced with SF and LP at two levels (5 and 10% by mass) without changing the FA content. The effect of PCM addition (5 and 10% by mass of fine aggregate) on thermal conductivity was studied further, using mixtures (referred to as P5 and P10 in Mixture ID) that demonstrated the best printability without any defects. Table 2 shows the proportions of different mixtures. Ternary blends containing OPC, FA, and SF or LP were proportioned at a fixed sand-to-binder ratio of 1.0 and mass-based water-to-binder ratio of 0.39–0.40, so that the mixtures were extrudable. These 3D printable concrete mixtures were prepared using a Hobart mixer. Firstly, all dry ingredients were mixed at 107 rpm for 2 min to get a homogeneous dry mixture, and then for 1 min after adding the required amount of water. Mixing was then stopped for 30 s and scrapped the bowl. Finally, the mixing was continued for another 2 min, and then 1 min at 198 rpm and 361 rpm, respectively. In the case of PCM-containing mixtures, the mixing procedure remains the same except that the PCM was added to the wet mixture in the end, and mixed at 107 rpm for only 1 min to avoid encapsulation breaking.

### 2.2. 3D Concrete Printing

**Figure 3:**
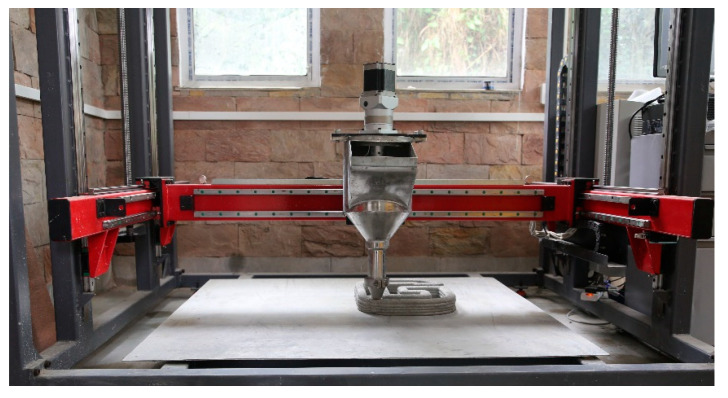
Lab scale 3 axis 3D concrete printer at IIT Guwahati, India.

**Figure 4:**
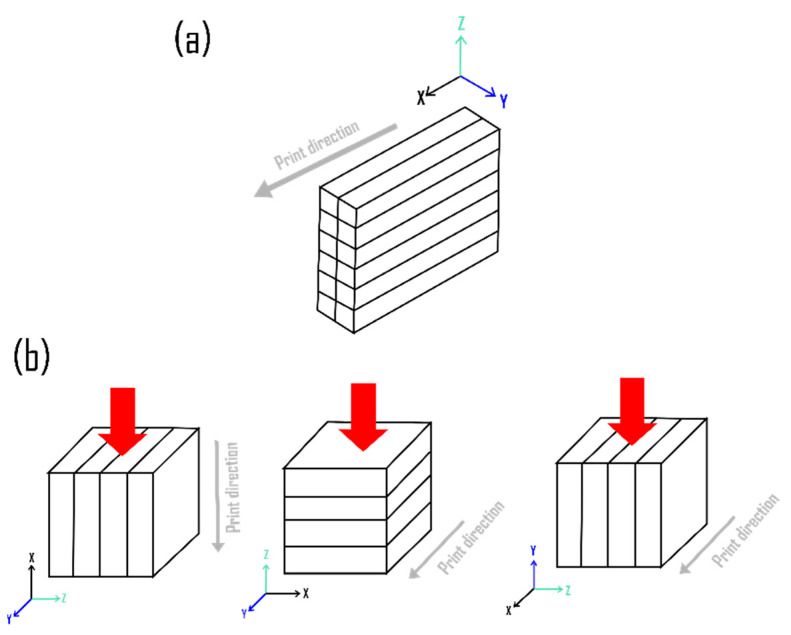
Illustration of (**a**) 3D printed beam and (**b**) the direction of loading (red arrow) on the extracted cubes during the compression test.

A 3 axis gantry type concrete printer was used in this study for printing the cementitious materials, as shown in Figure 3. As indicated in Figure 4a, specimens with dimensions of 300 × 100 × 70 mm (l × b × h) were printed directly from the 3D model. The printing parameters used in this study were layer height of 10 mm and printing speed of 80 mm/s.

### 2.3. Test Methods

**Figure 5:**
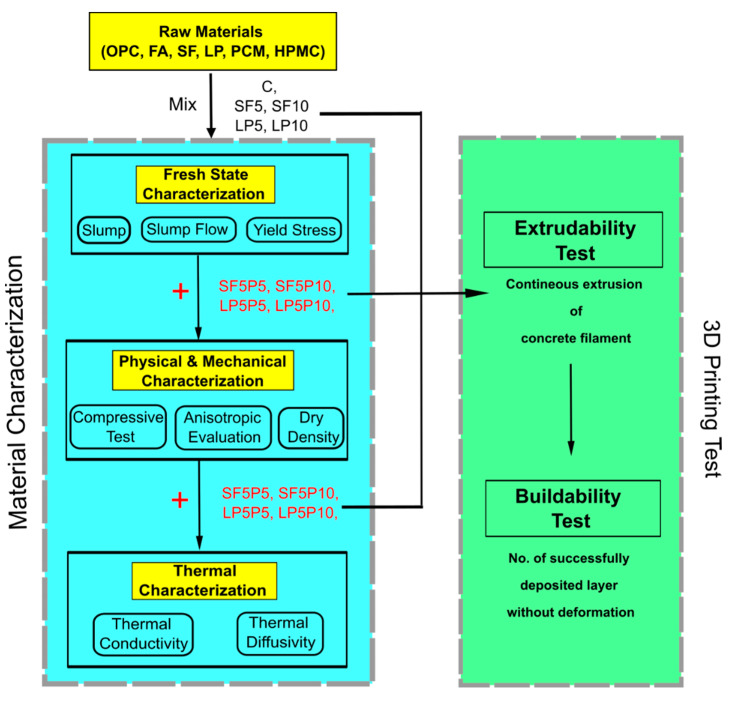
Flowchart of the test methods.

The methods employed in this study are described in this section and schematically depicted in Figure 5.

#### 2.3.1. Flow Table Test

The flowability of the materials was tested by a mini-slump cone, having an inside diameter of 70 mm at the top and 100 mm at the bottom, and a height of 60 mm. According to ASTM C1437-20 [33], the spread diameter of fresh mortar mixtures was measured after 25 drops of the flow table.

#### 2.3.2. Estimation of Yield Stress of Cementitious Materials

Concrete has a time-dependent material behavior, in which the yield stress increases as concrete matures in its fresh state. This phenomenon occurs as a result of the flocculation of cement particles, and the formation of an internal structure soon after mixing and while the mixture is at rest. Roussel and Coussot devised an analytical model [34] and presented dimensionless strain ($s^{\prime }$) and stress ($\tau_{c}^{'}$), as shown in Equations (1) and (2), respectively, to estimate the yield stress of coarse materials that depends on the three different flow regimes, namely, pure shear flow (h ≪ r), pure elongational flow (h ≫ r), and intermediate instance (h ≈ r) [34].

(1) $$s^{\prime }=\frac{s}{h_{o}}$$

(2) $$\tau_{c}^{'}=\frac{\tau_{c}}{\rho gh_{o}}\;$$

where *s* is the slump after demolding; *h_o_* is the initial height of the specimen; $\tau_{c}$ is the yield stress of the material; ρ is the density of the material; *g* is the acceleration due to gravity; $h_{o}$ is the specimen height; and r is the radius of the specimen after demolding.

Since all the mixtures generated in this study demonstrated pure elongational flow with $s^{\prime }$ ≪ 1, the authors utilized Equation (3) to calculate the elastic stress [34].

(3) $$\tau_{c}^{'}=\frac{1-s^{\prime }}{\sqrt{3}}$$

The slump and diameter of the specimen after demolding were measured using a 3D printed cylindrical mold with an internal diameter of 65 mm (3 mm thick) and a height of 131 mm. Another 3D printed cylindrical container with an interior diameter of 77 mm (3 mm thick) and a height of 105 mm was used to determine the density of the fresh mortar mixtures. The test procedure can be found elsewhere [35].

#### 2.3.3. Buildability Test

It should be noted that there is no standard quantification to measure buildability. In this work, a cylinder having a diameter of 200 mm was printed for all the mixtures, with a constant printing speed of 80 mm/s and an extrusion rate of 25.12 mL/s, to compare the maximum number of layers up to which the materials can be built without structural collapse. The maximum number of deposited layers before collapsing was recorded as a visual indicator for this test. A similar method is used commonly in the literature to measure the buildability of a 3D printed structure [36,37,38].

#### 2.3.4. Compressive Strength Test and Evaluation of Anisotropy

As shown in Figure 4a, a concrete block with dimensions of 300 × 100 × 70 mm (l × b × h) was printed, and then specimens having dimensions 50 × 50 × 50 mm were extracted from the block using a water-cooled, diamond saw cutter. The surface of the specimens was smoothened using a metal filer and the directions were marked appropriately. In addition, 50 × 50 × 50 mm size cubes were mold-cast in line with the ASTM C 109 standard [39] for performance comparison.

The specimens were subjected to compressive loads after 28 days of curing using a hydraulic controlled compression testing machine (CTM) having a capacity of 2000 kN, and the loading was applied monotonically until failure at 1000 N/s. The effect of SF and LP on mortar compressive strength was evaluated according to ASTM C109. To evaluate the anisotropic behavior of 3D printed structures [40], three cubes were tested in each direction (Figure 4b) and these findings were compared with the casting results. To further understand the effects of SF and LP on anisotropy induced by printing parameters, the anisotropic coefficient ($I_{3D}$) of printed specimens was determined using the empirical parameter given by Ye et al. [41] (see Equation (4)). Anisotropic coefficients in different directions ($I_{X}$, $I_{Y}$, and $I_{Z}$) can be found from Equation (5) [41].

(4) $$I_{3D}=(I_{X}+I_{Y}+I_{Z})/3$$

(5) $$I_{Direction}=\sqrt{{\left(f_{X}-f_{Direction}\right)}^{2}+{\left(f_{Y}-f_{Direction}\right)}^{2}+{\left(f_{Z}-f_{Direction}\right)}^{2}}/f_{Direction}$$

where ‘*Direction*’ represents the loading directions and $f_{Direction}$ represents the compressive strength corresponding to that direction.

#### 2.3.5. Density

After 28 days of curing, the specimens were surface dried and then the bulk densities (weight of the dried specimens divided by their known volume) of the specimens were calculated by taking the average of three cube specimens having dimensions 50 × 50 × 50 mm. For the printed component, 50 mm cubes (extracted from a 300 × 100 × 70 mm slab) were used for density measurement.

#### 2.3.6. Thermal Test

**Figure 6:**
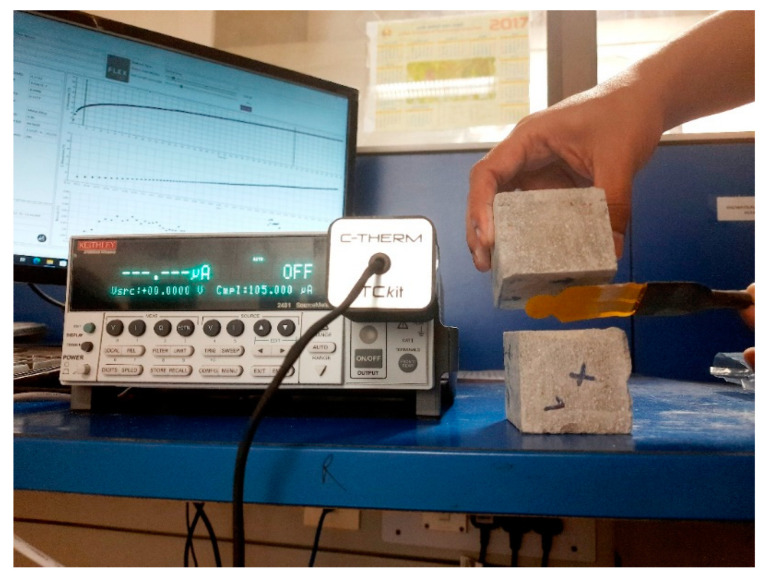
Thermal conductivity measurement of 3D printed cubes using transient plane source.

The C-Therm TC kit configured with the Transient Plane Source (TPS) technique was used for simultaneous measurements of thermal conductivity and thermal diffusivity of all specimens at room temperature and normal pressure. The TC kit has a measuring range of 0.03–60 W/mK with a precision of greater than 2%. The TPS technique comprises an electrically conducting pattern in the shape of a bifilar spiral that acts as a temperature sensor in the specimen [42]. As illustrated in Figure 6, the sensor is sandwiched between two cubes specimens measuring 50 × 50 × 50 mm. Electrical power is applied to the sensor’s spiral heating element, generating a voltage shift across the sensor element. The test timeframe was set at 40 s and the power level was set at 0.9 watts, based on the initial scouting run. The test findings were presented as a temperature vs. time graph in the software interface. Thermal property data such as thermal conductivity and thermal diffusivity are computed from that graph utilizing an iterative solution approach. Volumetric heat capacity (Cv) of the material was then calculated by dividing the thermal conductivity (K) by that of its diffusivity (α) value, as shown in Equation (6) [27].

(6) $$\mathrm{Cv}=\frac{K}{\alpha }$$

## 3. Results and Discussion

### 3.1. The Effect of Mix Design on Extrudability and Buildability

**Figure 7:**
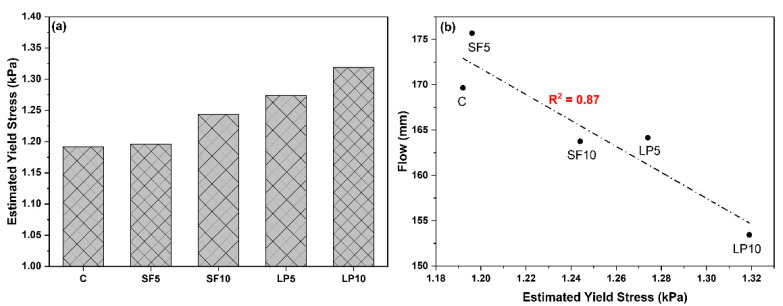
(**a**) Yield stress (**b**) correlation between estimated static yield stress and flow of printable mixtures.

Printability is defined as the ability of a mixture to extrude (extrudability) and maintain the structural integrity when built in layers (buildability) [43]. All the mixtures shown in Table 2 were found to be extrudable, due to their yield stress (see Figure 7a) value being greater than the minimum yield stress required for 3DCP. Figure 7b depicts the relationship between the estimated static yield stress, and flow of the mixtures. The control mixture “C” was found to have the lowest yield stress among all. Direct substitution of PC with SF up to 10% (SF 5 and SF10), significantly improved the yield stress of ternary mixtures at a fixed water to binder ratio, which is generally attributed to particle size of the SF. Due to its smallest particle size (among all binders), the SF can fill the spaces between relatively large cement grains (average size 10 μm) and increase the inter-particle interactions and friction, thus resulting in higher yield stress [35,40]. The yield stresses of SF-containing mixtures vary between 1.18 kPa and 1.25 kPa, which is similar to the extrudable mortars used in other studies. This high yield stress obtained using SF allows the material to sustain the load of its own weight, and weight of the subsequent layers, during 3D printing.

**Figure 8:**
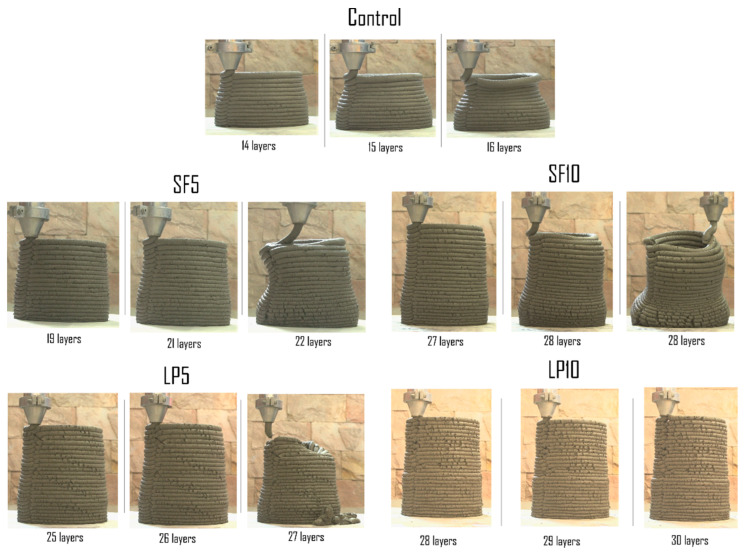
Comparison of buildability (in terms of number of layers) of cementitious mixtures.

On the other hand, mixtures containing 5% and 10% LP (LP5 and LP10) had more water demand, and hence the water to binder ratio was increased to 0.393 and 0.405, respectively, to make the mixtures extrudable. The yield stress of LP5 and LP10 mixtures were increased to 1.27 and 1.32 kPa, owing to the particle size distribution and coarser content of the LP above 100 µm [19,44,45]. A lower slump flow value was reported, corresponding to higher yield stress (see Figure 7b). The effect of LP addition on buildability is shown in Figure 8, which shows that the “C” mixture begins to elastically collapse at the 15th layer; whereas, the SF5 mixture retains its shape until the 21st layer and SF10 mixture until the 28th layer, giving nearly 35% and 92% improvement in buildability, respectively. Similarly, the maximum height of the 27th layer and 30th layer for LP5 and LP10 could be built, respectively. This could also be attributed to the higher structural build-up or flocculation rate in LP-containing mixtures. For example, the flocculation rate of concrete containing 10% limestone powder was approximately 1.32 times that of reference concrete [46]. Although the LP10 mix did not fail at the 30th layer, the printing was paused as the ‘surface cracks’ began to appear in the filament. Further, the increased hydration reaction, caused by LP presence and formation of more C-S-H and CH at early ages, is attributed to the higher buildability of mixtures containing LP [47]. These hydration products establish rigid bridges between mixture constituents, increasing the material’s yield stress [48]. This chemical reaction is primarily caused by the reaction of LP and alumina present in the binder, which results in the formation of carboaluminate. This reaction is accelerated when LP is combined with SCMs containing Al_2_O_3_ [47]. It is also important to note that the addition of PCM did not result in significant changes, in terms of maximum number of printed layers; therefore, in-depth investigations are needed to understand the influence of PCM on extrusion rheology and printable properties.

### 3.2. Compressive Strength Properties of 3D Printed Mixtures

**Figure 9:**
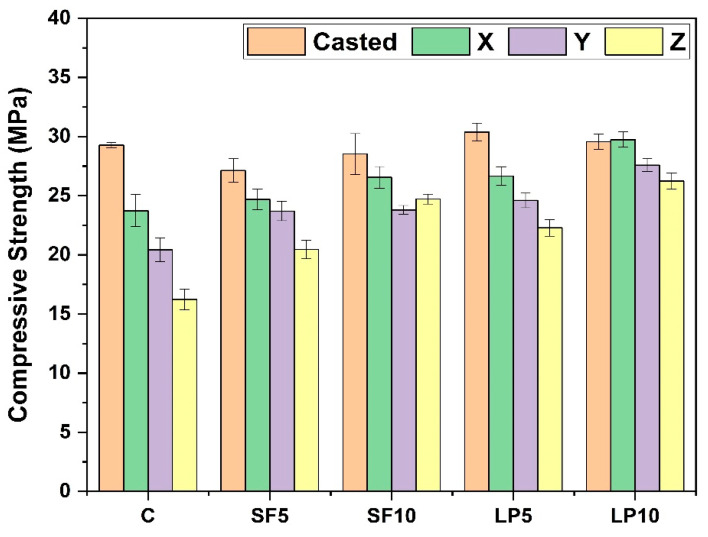
28-day compressive strength of cast and printed specimens (C = control specimen and X, Y, Z directions are meant for only 3D printed specimens).

The compressive properties of the 3D printed specimens were evaluated after 28 days of curing regimes. Figure 9 depicts the compressive strength of casted, as well as printed, specimens without PCM. The compressive strength of the printed specimens was found to be lower than the casted specimens for all the mixtures. Many researchers [40,49,50,51,52] have reported similar observations due to intrinsic printing characteristics. Strength variation in the 3D printed specimens, as a consequence of varying the contact area of the extruded filaments, was also noticed; meanwhile, the direction perpendicular to the printing direction exhibited the lowest compressive strength. The 28-day compressive strength of mortar specimens containing SF (SF5 and SF10) was found to be lower than that of the control. This finding is consistent with the previous study [53], which shows that the combination of SF and FA has a significant effect on the strength improvement at later ages (after 28 days), owing to the synergetic pozzolanic and micro filling effect [53]. It is the fly ash which governs the compressive strength property of specimens, when present in the ternary blended mixtures [54]. According to the literature, the impact of SF on mechanical characteristics is further influenced by the presence of aggregate [55] and other SCMs [53]. It should be noted that the SF10 mixture exhibited enhanced compressive strength (up to 20.8%), compared to the SF5 mixture, in the case of printed specimens.

**Figure 10:**
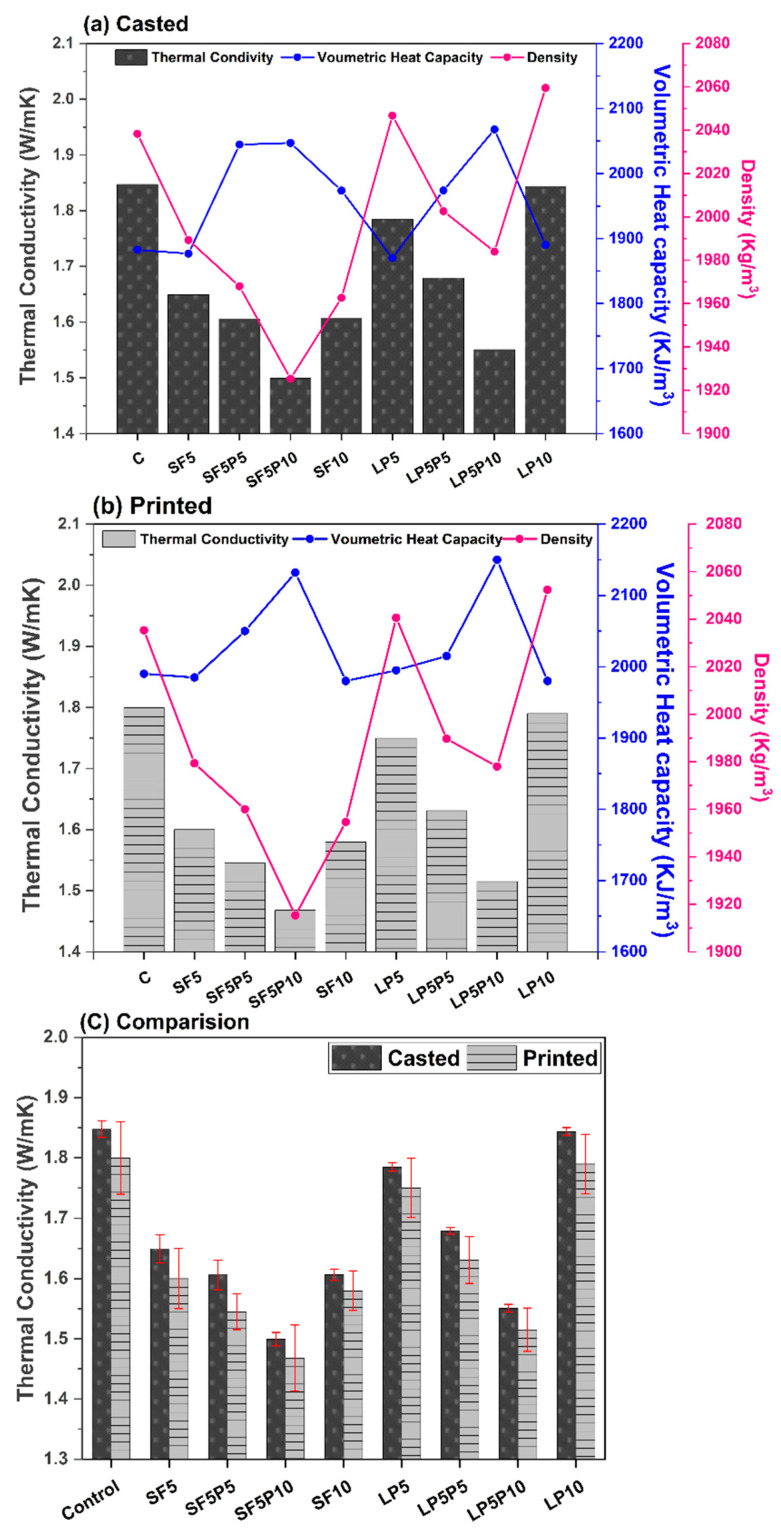
Thermal conductivity, volumetric heat capacity and density of (**a**) casted, (**b**) 3D printed specimens, and (**c**) their comparative analysis.

In contrast, the LP-containing mixtures resulted in a maximum 4% increase in compressive strength due to the filler effect, which is substantiated by the increase in density of the mixtures (see Figure 10a,b). The effect of LP on the properties of cementitious mixtures is also affected by the replacement level. Replacement levels beyond the optimum dosage may reduce mechanical properties due to dilution, but at the optimum dosage the effect is negated by the combined action of filler effect and heterogeneous nucleation [49,50,52].

**Figure 11:**
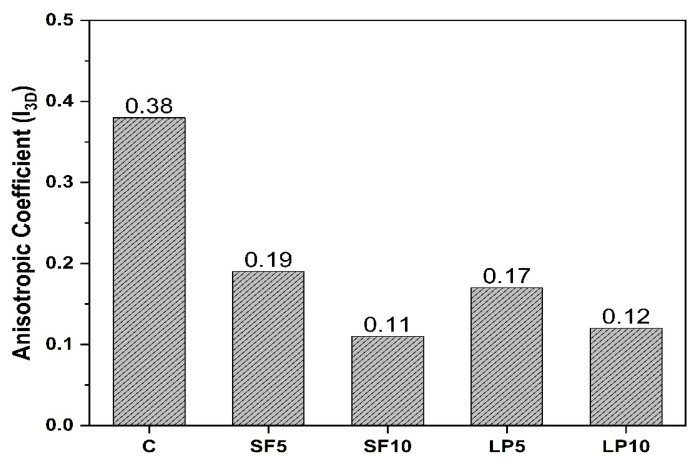
Compressive strength anisotropic coefficient of printable mixtures.

Figure 11 compares the anisotropic coefficients (*I_3D_*) of printed specimens containing SF and LP at various replacement levels to control specimens. The *I_3D_* value of compressive strength changes from 0.19 to 0.11 and 0.17 to 0.12 for SF and LP mixtures, respectively, whereas it is 0.36 for the control mixture. The anisotropic coefficient of the control specimen lies in the value range (0.25–0.47) reported in the previous research [41,51]. It is evident from the results that the presence of SF and LP reduced the anisotropy caused by the printing process. This decrease in anisotropic coefficient may be attributed to the formation of higher hydration products at layer interfaces, resulting in stronger interlayer properties [56,57,58]. It can thus be inferred from this experimental study, that usages of SCMs not only improve the rheological and mechanical performance [59], but also minimize mechanical anisotropy generated by the printing process. Though several studies in the literature [43] claim that 3D printed components have improved mechanical characteristics, this needs thorough material design and process parameter optimization.

**Figure 12:**
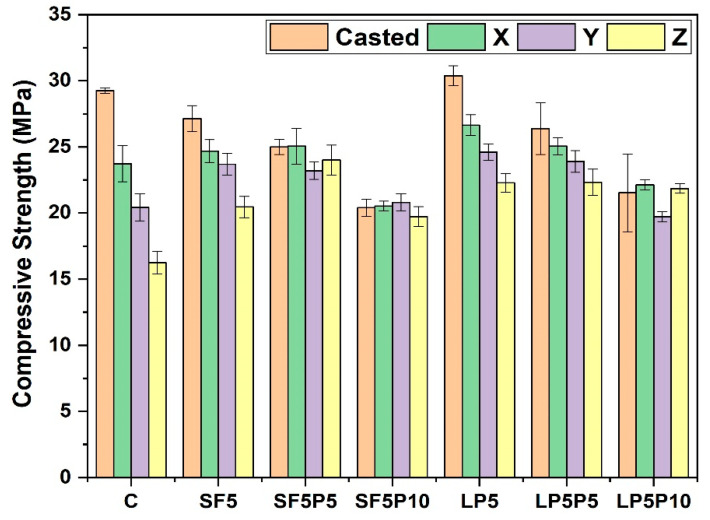
28-day Compressive strength of cast and printed specimens with and without PCM.

The 28-day compressive strength of printed mixtures with varied PCM dosages (Figure 12) was found to be lower than the control and corresponding mixtures without PCM. This holds true for both casted as well as printed specimens of all mixes, and the decrease in compressive strength is more significant at higher dosages of PCM. While the SF and LP-containing mixtures with PCM (5 and 10% by mass of fine aggregate) are compared to their counterparts without PCM, the compressive strength of casted specimens was reduced by 7.8–24.8% and 13.2–29.1%, respectively. Brooks et al. [28] found the same trend in casted compressive strength in a previous study. This might be owing to the PCM acrylic shell’s poor interfacial bond with the surrounding binder phase [60] and the specimens’ decreased bulk density, as seen in Figure 10a,b.

### 3.3. Thermal Properties of 3D Printed Mixtures

It was found, from Figure 10, that upon substitution of OPC with SF, the thermal conductivity and thermal diffusivity of the mixtures decreased, making the volumetric heat capacity of the mixtures higher. The decrement of thermal conductivity was almost 12% for SF10 (both casted and printed), whereas the increment in volumetric heat capacity was only 4% in comparison to the control specimen; this is in line with the finding from the literature [23]. Given that crystalline silica has roughly 15 times higher thermal conductivity than amorphous silica [23], it is thus natural for concretes containing amorphous silica to have reduced conductivity [61]. Hence, higher air content, as well as the amorphous structure of SF, are most likely to be responsible for decreased density and thermal conductivity of the SF modified mixtures. In contrast, substitution of the PC with LP increased the density and strength slightly but did not significantly affect the thermal conductivity and heat capacity of the mixtures. Similar results have also been reported by Zhou et al. mentioning a very slight increase in the thermal properties (thermal conductivity, diffusivity) of the mixtures, with up to 50 wt.% substitution of LP in cementitious mixture [62].

As SF5 and LP5 mixtures showed the most promising properties for printability, the effect of PCM addition was studied on it too. When compared to SF5 and LP5 mixtures, it was noticed that SF5P10 and LP5P10 mixtures have lower thermal conductivity (9% for SF5P10 and 13% for LP5P10) and higher heat capacity (9% for SF5P10 and 10% for LP5P10), making them thermally active to insulate and store heat whenever needed. This is due to PCM’s lower thermal conductivity (0.21 W/mK), compared to concrete material (1.5 W/mK) and its larger thermal mass, which allows the heat to be captured without raising the specimens’ sensible temperature. This effect is also reflected by the drastic downward shift in density and strength as shown in Figure 10a,b and Figure 12, respectively. Similar results were seen by Brookes et al. [28], who ascribed this impact to the formation of a weak interfacial zone around the PCM particle, resulting in poor stiffness and bonding strength, and hence increased resistance in heat transport. It is also important to note that the low thermal conductivity and increased heat capacity were noticed for both casted and printed specimens as shown in Figure 10a,b indicating no adverse effect of extrusion on the encapsulated PCM particles during printing.

The thermal conductivity of all mixtures (both casted and printed) was found to be in the range of 1.49 to 1.84 W/mK. It was also found that the average thermal conductivity of printed specimens is slightly lower than casted specimens for all the mix designs, as shown in Figure 10c. This difference is due to the decrease in density of the printed elements, as a result of reduced compaction pressure during 3D printing [24].

### 3.4. Correlation between Mechanical and Thermal Properties

The majority of studies in the literature highlight the correlation between thermal conductivity and density of cement-based materials, and there are relatively few studies that can predict the thermal conductivity based on the other physical and mechanical characteristics of the material [63]. In a recent study by Shafigh et al. [63], a positive correlation between compressive strength and thermal conductivity, and negative correlation with porosity of the mortar, was found for cement mortars. However, in this study despite having higher compressive strength, the thermal conductivity of the SF10 mix was lower than SF5. This can be explained by the presence of a high proportion of amorphous silica in the SF, which has lower thermal conductivity. Furthermore, the lower density of the SF, compared to the PC, material and dry density of the mortar (both casted and printed) could also explain this behavior. A similar trend can be seen in the mixtures containing LP at low dosages (5%). Despite having lower density (than PC), the LP produced comparable or slightly higher thermal conductivity than the control mix at higher LP dosages (10%). This effect can be explained by an increase in the dry density of the mortar due to increased particle packing induced by the presence of LP (pertaining to its high fineness compared to OPC and SF). However, the replacement of fine aggregate with PCM has resulted in lower thermal conductivity, due to the reduced compressive strength and density, even with the mixtures containing SF and LP. Hence, it is reasonable to replace the PC with SF or LP (at lower dosages) with inclusion of the PCM.

## 4. Conclusions

This paper has elucidated the influence of silica fume and limestone powder on the 3D printability, compressive strength, and thermal conductivity of cementitious materials. The substitution of cement with silica fume and limestone powder (up to 10%) improved the buildability due to higher static yield stress, without affecting the extrudability of the mixtures. The compressive strengths were found to be lower (2–7%) for silica fume modified specimens, however limestone addition resulted in 4% and 1% increases in the strength for 5% and 10% replacement levels, respectively, compared to the control blend. It was observed that extrusion-based 3D printing produced anisotropic and lower mechanical properties, which is likely due to weak interlayer bonding and low density of the extruded layers. Usage of silica fume and limestone powder in the printable mixtures proved to reduce the compressive anisotropy. The thermal conductivity results revealed comparable thermal performance of 3D printed specimens, with respect to casted specimens. The inclusion of PCM improved the thermal performance of both SF and LP mixes, as evaluated by a decrease in thermal conductivity, and an increase in volumetric heat capacity, respectively. Further investigation is needed to understand the effect of PCM on the rheology and printability of cementitious mixtures.

## Figures and Tables

**Table 1:** Chemical composition of binders.

Components of the Binders	Chemical Composition (% by Mass)							Specific Gravity
	SiO_2_	Al_2_O_3_	Fe_2_O_3_	CaO	MgO	SO_3_	LOI	
PC	19.62	5.62	5.33	61.24	0.88	2.60	2.06	3.15
FA	56.50	26.16	7.24	7.50	1.60	0.55	2.53	2.08
SF	87	-	-	-	-	-	2.00	2.17
LP	2.88	1.25	1.18	43.82	2.73	0.32	45.76	2.15

**Table 2:** Details of the mixture proportion.

Mix ID	Mass Fraction of Binders				PCM (wt.% of Fine Aggregate)	VMA (wt.% of Binder)	Sand-to-Binder Ratio (s/b)	Water-to-Binder Ratio (w/b)
	OPC	FA	SF	LP				
C	0.80	0.2	0	0	0	0.1	1.0	0.39
SF5	0.75	0.2	0.05	0	0			0.39
SF5P5	0.75	0.2	0.05	0	5			0.39
SF5P10	0.75	0.2	0.05	0	10			0.39
SF10	0.70	0.2	0.10	0	0			0.39
LP5	0.75	0.2	0	0.05	0			0.393
LP5P5	0.75	0.2	0	0.05	5			0.393
LP5P10	0.75	0.2	0	0.05	10			0.393
LP10	0.70	0.2	0	0.10	0			0.405

## Data Availability

Data will be made available on request.
